# Draft Genome Sequence of Isolate *Staphylococcus aureus* LHSKBClinical, Isolated from an Infected Hip

**DOI:** 10.1128/genomeA.00336-15

**Published:** 2015-04-30

**Authors:** Laurence H. Stipetic, Graham Hamilton, Matthew J. Dalby, Robert L. Davies, R. M. Dominic Meek, Gordon Ramage, David G. E. Smith, Karl E. V. Burgess

**Affiliations:** a Institute of Infection, Immunity and Inflammation, College of Medical, Veterinary and Life Sciences, University of Glasgow, Glasgow, United Kingdom; b Glasgow Polyomics, University of Glasgow, Glasgow, United Kingdom; c Institute of Molecular Cell and Systems Biology, University of Glasgow, Glasgow, United Kingdom; d Glasgow Dental School, School of Medicine, College of Medical, Veterinary and Life Sciences, University of Glasgow, Glasgow, United Kingdom; e Department of Orthopedics, Southern General Hospital, Glasgow, United Kingdom; f Moredun Research Institute, Edinburgh, United Kingdom

## Abstract

We report here the genome sequence of a clinical isolate of *Staphylococcus aureus* from an orthopedic infection. Phenotypically diverse *Staphylococcus aureus* strains are associated with orthopedic infections and subsequent implant failure, and some are highly resistant to antibiotics. This genome sequence will support further analyses of strains causing orthopedic infections.

## GENOME ANNOUNCEMENT

Members of the genus *Staphylococcus* are primarily responsible for orthopedic infections after arthroplasty surgery (1–3) and subsequent implant failure (4). When isolated from orthopedic cases (2), *Staphylococcus aureus* is considered a primary medical concern due to the emergence of antimicrobial-resistant strains (5) and the ability of *S. aureus* to attach to surfaces and excrete an extracellular matrix to form biofilms (1, 6). Biofilm formation has been shown to exacerbate antibiotic resistance (7, 8), providing a protective environment for the indwelling bacteria. Biofilm cells display an altered gene expression profile (9) and metabolism in contrast to their planktonic counterparts (10–12). Differences occur in biofilm capability between *S. aureus* strains, with certain strains forming biofilms more readily than others (6). A number of genes have been found to be important in the formation of a biofilm by *S. aureus* (9).

*S. aureus* LHSKBClinical was isolated at the Southern General Hospital Microbiology laboratories (Glasgow, United Kingdom) from pus aspirated from the hip of a 75-year-old female. Speciation was confirmed on the basis of a positive Staph Xtra latex test (Pro-Lab Diagnostics) and growth on bioMérieux chromID agar (SAID). Genomic DNA was prepared from a brain-heart infusion (BHI) broth culture using the DNeasy blood and tissue kit (Qiagen) according to the manufacturer’s instructions, utilizing the protocol for Gram-positive cell lysis (13). Sequencing was carried out using the Illumina MiSeq system, employing 300-bp paired-end sequencing. The reads were trimmed of Illumina adapter sequences and low-quality bases, and then *de novo* assembled and scaffolded using the CLC Genomics Workbench version 7.5.1 (Qiagen). Assembled scaffolds totaled 24, consisting of a total of 2,762,774 bp with an *N*_50_ of 587,246 bp and an A-T content of 67%, similar to other sequenced strains of *S. aureus* (14, 15). Assembled scaffolds were annotated using the Rapid Annotations using Subsystem Technology (RAST) resource (16, 17). The genome sequence was predicted to encode 2,566 proteins.

Interrogating http://saureus.mlst.net (18–20) showed that this strain did not match any existing sequence type, and alignment of assembled scaffolds using NCBI BLAST searches (21) and Mauve genome analysis software (22) identified regions of heterogeneity compared against other previously sequenced *S. aureus* genomes.

Genes encoding *icaA* and *icaD*, polysaccharide intracellular adhesion proteins associated with *S. aureus* biofilm formation (9), were found. Furthermore, *sarA*, another gene implicated in biofilm formation by *S. aureus* (23, 24), was also found in the genome. Moreover, *fnbB* was found in the genome, perceived as a possible genetic marker for biofilm formation (25). Additionally, several genes encoding exotoxins or superantigens were found in the genome, including several associated with super-antigen-encoding *S. aureus* pathogenicity islands (SaPI), a virulence factor responsible for increased pathogenicity (26, 27).

The genome shows a novel sequence type for this isolate and provides a reference genome for *S. aureus* from orthopedic infections, both aspects of pertinence to further comparative studies of *S. aureus* pathogenicity.

### Nucleotide sequence accession numbers. 

This whole-genome shotgun project has been deposited at DDBJ/EMBL/GenBank under the accession number JZAL00000000. The version described in this paper is version JZAL01000000.
